# Data on investigation of hypoglycemic, anti-cholesteremic, in vivo antioxidant and pancreatic beta cell protective effect of *Putranjiva roxburghii* Wall bark in streptozotocin-induced diabetic rats

**DOI:** 10.1016/j.dib.2018.04.124

**Published:** 2018-05-02

**Authors:** Kedar Kalyani Abhimanyu, Chaudhari Sanjay Ravindra, Rao Srinivasa Avanapu

**Affiliations:** a Department of Pharmacognosy, Progressive Education Society's Modern College of Pharmacy, Sector-21, Yamunanagar Nigdi, Pune 411044, Maharashtra, India; b Jawaharlal Nehru Technological University (JNTU), Hyderabad, Andra Pradesh 500072, India; c Rasiklal M. Dhariwal Institute of Pharmaceutical Education and Research, Pune, India; d Bhaskar Pharmacy College, Yeknapally, Moinabad (Mandal) R.R (Dt), Hyderabad 500075, India

**Keywords:** EAPR, ethyl acetate extract of *Putranjiva roxburghii* Wall barks, BSI, botanical survey of India, GC-MS, gas chromatography and mass spectrometry, STZ, streptozotocin, i.p, Intraperitoneal, o.i.d, once in day, SOD, superoxide dismutase, CAT, catalase, IAEC, Institutional Animal Ethical Committee, CPCSEA, Committee for purpose of control and supervision of experimentation on animals, OECD, Organisation for economic co-operation and development, ANOVA, Analysis of variance, ROS, Reactive oxygen species, *Putranjiva roxburghii* Wall barks, Streptozotocin, Antihyperglycemic, Anti-cholesterolemic, Antioxidant

## Abstract

The data existing in this article are associated to the antidiabetic activity of ethyl acetate extract of *Putranjiva roxburghii* Wall barks (EAPR) in streptozotocin (STZ) induced diabetic rats at a dose of 250 & 500 mg/kg by oral route for 21 days. The phytochemical screening of the extract was carried out by gas chromatography and mass spectrometry. Diabetes was induced by streptozotocin (50 mg/kg; i.p), EAPR (250 & 500 mg/kg; b.wt) and standard Insulin (6 IU/animal; subcutaneous; o.i.d) were administered to the diabetic rats. Body weight and blood glucose were estimated weekly. Cholesterol, SOD and CAT were estimated in the blood serum on 21 days of the investigation period. Oral administration of EAPR (500 mg/kg) significant rises in the body weight, decrease in the blood glucose and total cholesterol and restore function of SOD and CAT enzymes (*P* < 0.05). Current data were also supported by histological study, necrosis was observed in the diabetic rat pancreas; however, necrosis was less observable in treated groups. These findings reveal that an ethyl acetate extract of *Putranjiva roxburghii* Wall barks shows antihyperglycemic, anti-cholesterolemic, antioxidant and improved the cell density of β-cells of islets of Langerhans in diabetic rats.

## Specifications Table

**Table:** 

**Subject area**	*Pharmacy*
**More specific subject area**	*Antidiabetic activity of medicinal plant*
**Type of data**	*Table, text file, graph, figure*
**How data was acquired**	*Gas chromatography and mass spectrometry (GC-MS) was performed on GCMS QP2010 Ultra (Shimadzu) including Mass Spectrometer equipped with EI source, fitted with Rtx-5MS capillary column (l-30 m T-0.25 µm × Dia-0.25 mm)*
**Data format**	*The results were expressed as mean SEM from six animals. The results were subjected to statistical analysis by using one way ANOVA followed by Tuckey's test p < 0.05 was considered to be statistically significant.*
**Experimental factors**	*Ethyl acetate extract of the bark of the Putranjiva roxburghii Wall was prepared by soxhlet extraction assembly*.
**Experimental features**	1. *The acute toxicity data for ethyl acetate extract of bark was performed by using female Wistar rats followed by OECD Guidelines No. 423.* [6] 2. *Type-1 diabetes was induced in 48 rats by i.p injection of STZ (50 mg/kg) freshly prepared in 0.1 M sodium citrate buffer, pH 4.5. Seven days behind STZ administration, diabetes was confirmed by the presence of hyperglycemia* [7]. 3. *Rats were divided into five groups of six rats (n = 6) each. The Group I and II served as normal control and diabetic control, respectively receives saline (0.2 ml oral). Group III serves as a standard and Group IV and V were treated with ethyl acetate extract of the bark of the Putranjiva roxburghii Wall at different doses.*
**Data source location**	*Department of Pharmacognosy,*
	*Progressive education society's Modern college of pharmacy,*
	*Sector-21, Yamunanagar Nigdi, Pune-411044, Maharashtra*
**Data accessibility**	*All data are given along with the article and will be available for education and research work.*

## Value of the data

• The antidiabetic data noted in the *Putranjiva roxburghii* Wall are recognized to a) stimulation of in vivo antioxidant enzyme (SOD and CATALASE) b) regeneration of β-cells and c) stimulation of insulin liberate.
• The biological activity revealed by active phytoconstituents and extracts of *Putranjiva roxburghii* gives a considerable get through in the scheming of diabetes and its connected difficulty.
• Therefore, enhance in the nutritional ingestion of this plant species will put in innovative scope in the managing of diabetes.

## 1 Data

**Fig. 1:**
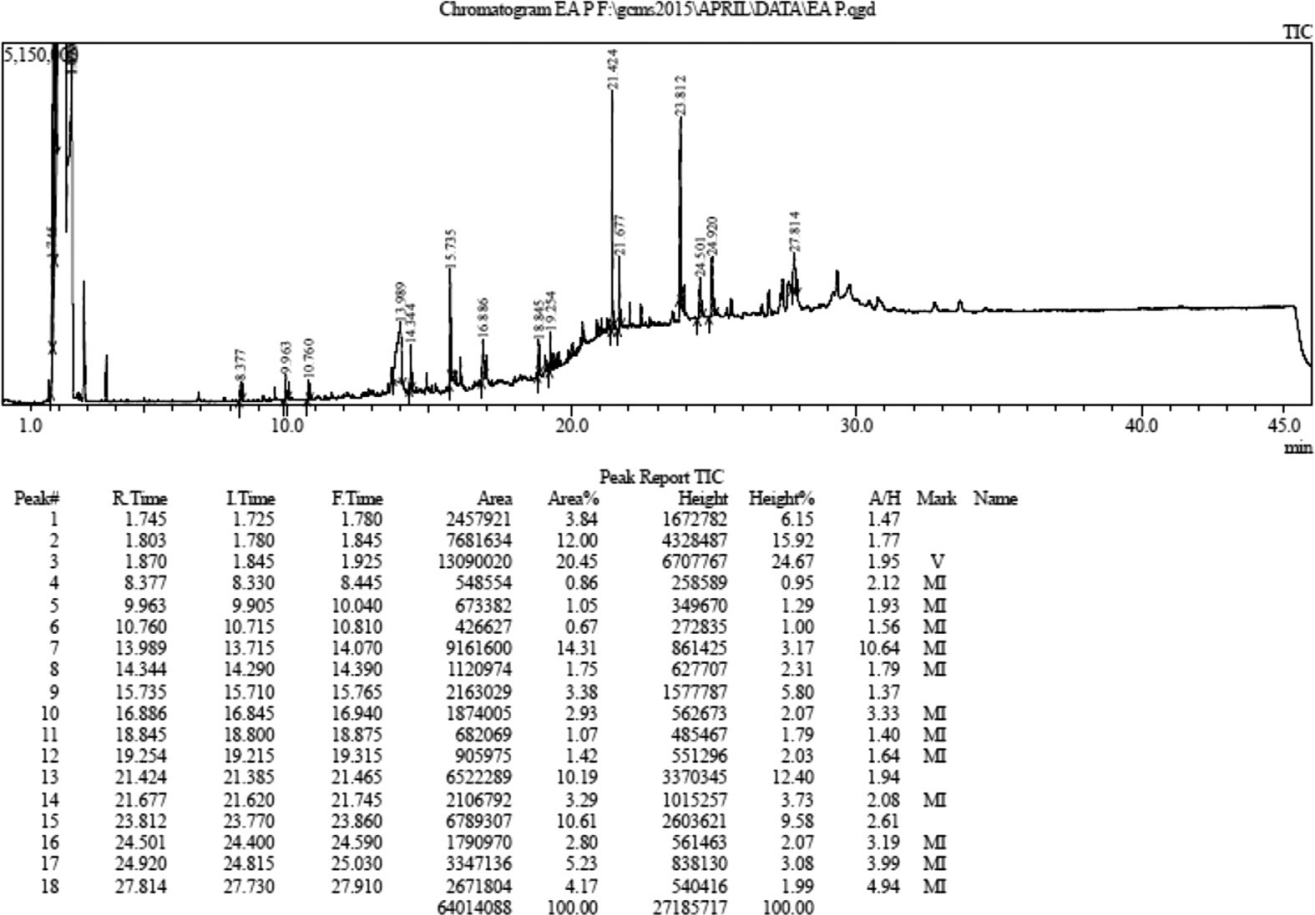
Gas chromatogram and mass spectrometry spectra of ethyl acetate extract of bark of *Putranjiva roxburghii* Wall (EAPR).

**Fig. 2:**
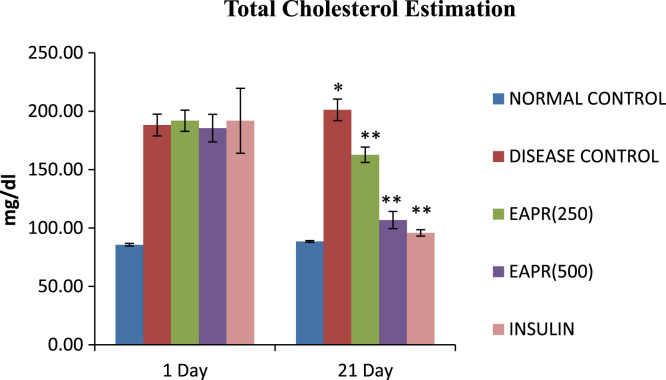
Effect of EAPR on cholesterol level in diabetic rats. The data are expressed as mean ± S.E.M.; *n = 6* in each group. ^*^p < 0.05, significant increase in cholesterol level as compared to normal control. ^**^p < 0.05, significant decrease in cholesterol level as compared to diabetic control.

**Fig. 3:**
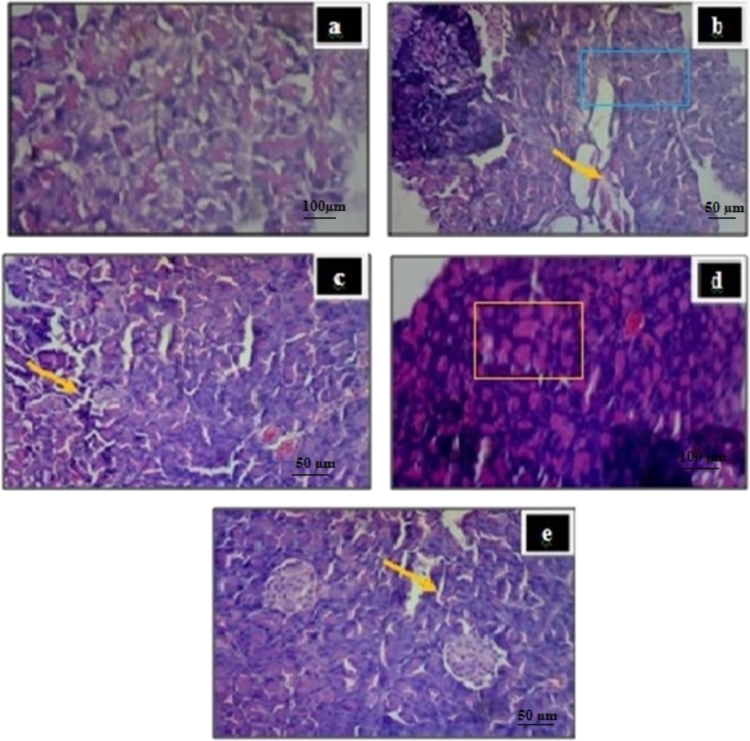
Histological changes of rat pancreas of islets of Langerhans. a) Non diabetic normal histological structure of rat pancreas showing normal islet. b) Diabetic control rat showing irregular cells and necrosis of cell destruction of ß-cells (indicated by the arrow and box). c) EAPR (250 mg/kg) showed destruction of ß-cells indicated by arrow. d) EAPR (500 mg/kg) showed increased cell size (indicated by colored box) by slight regeneration of β-cells were seen when compare with diabetic control. e) Insulin treated rat pancreas showing the normal density of the islet of β-cells along with few areas showing necropsy indicated by arrow.

The present data focus on the antidiabetic activity of ethyl acetate extract of *Putranjiva roxburghii* Wall barks in streptozotocin (STZ) induced diabetic rats. The data on chemical composition of ethyl acetate extract of bark *Putranjiva roxburghii* Wall by gas chromatography and mass spectrometry are shown in Fig. 1 and Table 1. The information regarding change in body weight, fasting blood glucose level, total cholesterol and in vivo antioxidant enzyme in diabetic rat during the experimental period are presented in Tables 2, 3, Fig. 2 and Table 4 respectively. Data regarding histological changes of rat pancreas of islets of Langerhans are shown in Fig. 3.

**Table 1:** Chemical composition of EAPR by gas chromatography and mass spectrometry chromatogram.

Peak, ID	Name and structure of compound	Retention time	Area %	Compound identified	Molecular weight
1	1,2,3 Propanetriol, 1-acetate	8.377	1.35	C_5_H_10_O_4_	134
2	Glycerol1, 2 diacetate	9.963	1.65	C_7_H_12_O_5_	176
3	Ethanone, 1-(2-hydroxy-5-methylphenyl)	10.76	1.05	C_9_H_10_O_2_	150
4	3-o methyl-d-glucose	13.98	22.46	C_7_H_14_O_6_	194
5	4-(1E)-3-hydroxy-1-propenyl)-2-methoxyphenone	14.34	2.75	C_10_H_12_O_3_	180
6	Pentadecanoic acid	15.73	5.30	C_16_H_32_O_2_	256
7	9-Hexadecenal	16.88	4.59	C_16_H_30_O	238
8	Pyrimidine, 5-heptyl-2 [4-(octyloxy) phenyl	18.84	1.67	C_25_H_38_N_20_	382
9	Bis (2-ethylhexyl) phthalate	19.25	2.22	C_24_H_38_O_4_	390
10	Friedelin	21.42	15.99	C_10_H_50_O	426
12	Friedo-2, 3secoleanane-2,3dioic acid, dimethyl ester	23.81	16.65	C_32_H_54_0_4_	502
13	β-Sitosterol	24.50	4.39	C_29_H_50_O	414
14	Ergosterol	24.92	8.21	C_28_H_48_O_4_	448
15	17-(1-5-dimethyl-3-phenyl-4-enyl)	27.81	6.55	C_36_H_54_O_5_	534

**Table 2:** Change in body weight of rats during the experimental period of 21 days.

Groups	Body weight (g)			
	0 day	7 day	14 day	21 day
Control	245.33 ± 4.75	253.83 ± 4.07	262.67 ± 3.21	273.67 ± 4.72
Diabetic control	257.83 ± 4.96	174.83 ± 4.03*	169.50 ± 4.49	163.67 ± 5.39
DC + EAPR (250)	243.83 ± 3.20	163.50 ± 4.10	169.17 ± 3.75	178.00 ± 3.48
DC + EAPR (500)	249.17 ± 4.80	157.83 ± 5.40	174.83 ± 6.70	197.83 ± 11.15**
DC+ Insulin (6 IU)	254.67 ± 5.31	174.83 ± 4.44*	193.00 ± 3.69	227.33 ± 13.89**

The data are expressed as mean ± S.E.M.; *n* = 6 in each group.

* *p* < 0.05, significant decrease in body weight as compared to weight on day 0.

** *p* < 0.05, significant increase in body weight as compared to weight on day 0.

**Table 3:** Effect on fasting blood glucose level in diabetic rats.

Treatment and dose	Blood glucose concentration in mg/dl		
	7 day	14 day	21 day
Control	93.67 ± 1.15*	93.83 ± 0.75	95 ± 1.24
Diabetic control	470.00 ± 9.31*	486.17 ± 9.21*	498.83 ± 8.54*
DC + EAPR (250)	452.17 ± 9.06*	324.50 ± 6.55**	272.00 ± 4.62**
DC + EAPR (500)	452.17 ± 9.06*	224.67 ± 7.37**	194.17 ± 5.80**
DC + Insulin (6 IU)	485.67 ± 27.84*	163.00 ± 2.70**	102.33 ± 5.36**

The data are expressed as mean ± S.E.M.; *n* = 6 in each group.

* *p* < 0.05.

**Table 4:** Effect of EAPR on antioxidant enzymes in diabetic rats.

Treatment and dose	Antioxidant enzyme	
	SOD (Unit/ml)	CATALASE (Ku/l)
Control	21.27 ± 1.41	0.34 ± 0.00
Diabetic control	12.66 ± 0.27*	0.20 ± 0.00*
DC + EAPR (250)	14.20 ± 0.40**	0.23 ± 0.01**
DC + EAPR (500)	18.79 ± 0.29**	0.29 ± 0.00**
DC + Insulin (6 IU)	19.51 ± 0.24**	0.32 ± 0.01**

The data are expressed as mean ± S.E.M.; *n* = 6 in each group.

* *p* < 0.05, significant decrease in SOD and CATALASE enzyme level as compared to normal control.

** *p* < 0.05, significant increase in SOD and CATALASE enzyme level as compared to diabetic control.

## 2 Experimental design, materials and methods

### 2.1 Plant collection and extraction

The trunk bark material of fully grown tree of the *Putranjiva roxburghii* Wall was collected from Khadki region of Pune district Maharashtra, in June 2014. The taxon is authenticated from Botanical Survey of India, Pune (voucher number BSI/WRC/Cert./2014 and collection no. KKA 01).

### 2.2 Extraction and phytochemical screening by gas chromatography and mass spectrometry

Ethyl acetate extract of the bark of the *Putranjiva roxburghii* Wall was prepared by soxhlet extraction assembly and the yield was 7.5% w/w use for phytochemical analysis. Gas chromatography and mass spectrometry (GC-MS) was performed on GCMS QP2010 Ultra (Shimadzu) including Mass Spectrometer equipped with EI source, fitted with Rtx-5MS capillary column (*l*-30 m *T*-0.25 µm × Dia-0.25 mm). Ultra-high purity helium was used as the carrier gas at a constant flow rate of 1.0 ml/min. The ionizing energy was 70 eV. The oven temperature was programmed from 50 °C (hold for 3 min) to 310 °C at a rate of 15 °C/min. All data were obtained by collecting the full-scan mass spectra within the scan range 8–45 amu. The identification of chemical compounds present in the ethyl acetate extract was carried out based on retention time which noted on gas chromatogram. However, the ethyl acetate extract of bark of *Putranjiva roxburghii* Wall reveals the presence of 15 phytoconstituents from this friedelin, sitosterol, ergosterol are in higher quantity as specified in Fig. 1 and Table 1. It means bark part of this plant is a rich source of steroids and terpenoids.

### 2.3 Chemicals and reagents

Streptozotocin was purchased from Sisco Research laboratories Pvt. Ltd. Insulin was purchased from Lantus Solostar of Sanofi India Limited. The biochemical data analysis was carried out by using standard diagnostic kits of span diagnostics, Ahmedabad, India. Other chemicals and reagents used in the study were of high purity.

### 2.4 Animals

Female Wistar rats weighing about 225–275 gm were obtained from. The data protocol was approved by the IAEC (Institutional Animal Ethical Committee) of CPCSEA (Committee for the purpose of control and supervision of experimentation on animals) with reference no. MCP/IAEC/167/2015, dated: 31/10/2015.

### 2.5 Acute toxicity studies (OECD [6])

The acute toxicity data for ethyl acetate extract of bark was performed by using female Wistar rats followed by OECD Guidelines No. 423, where different doses (30–2000 mg/kg of body weight) of ethyl acetate extracts were administered orally. Acute oral toxicity studies show that the nontoxic effect of ethyl acetate extract of the bark of the *Putranjiva roxburghii* Wall. Extracts were found to be safe in doses up to the 2000 mg/kg b.w. above the dose of 2000 mg/kg b.w., animal shows the signs as convulsions, weakness, and dizziness, loss of appetite, tremor and finally death.

### 2.6 Streptozotocin induced diabetes

Type-1 diabetes was induced in 48 rats by i.p injection of STZ (50 mg/kg) freshly prepared in 0.1 M sodium citrate buffer, pH 4.5 [1]. During the first 24 h of diabetes induction, STZ-treated animals were allowed to drink 5% glucose solution to overcome drug-induced hypoglycemia. After one week of STZ administration, diabetes was confirmed by the presence of hyperglycemia. STZ-treated animals showed fasting blood glucose less than 400 mg/dl was discarded [2].

### 2.7 Experimental design

Rats were divided into five groups of six rats (*n* = 6) each. The group I and II served as normal control and diabetic control, respectively receives saline (0.2 ml oral). Group III serves as a standard and was treated with Insulin (6 IU/animal; subcutaneous; once in a day). Group IV and V were treated with ethyl acetate extract of the bark of the *Putranjiva roxburghii* Wall at a dose of (250 mg/kg and 500 mg/kg per oral once in a day for 21 days). Body weight of each animal was monitored weekly during the period of the study the outcomes are shown in Table 2. Fasting blood glucose measured on day 7, 14 and 21 using a Glucose measurement kit by GOD POD method the consequences are presented in Table 3. Total cholesterol in serum was measured at 1 and 21 days [3] observations are made known by Fig. 2.

### 2.8 in vivo antioxidant activities

At termination on day 21, blood was collected using cardiac puncture and centrifuged at 7500 RPM for 10 min to obtain serum. Superoxide dismutase (SOD) activity was determined by the reported method [4]. Catalase (CAT) activity was determined according to the report method by Goth [5]. The outcome of this data is shown in Table 4.

### 2.9 Histopathology of the pancreas of streptozotocin induced diabetic rats

On the 21 days of the study, the animals were sacrificed. The pancreas was dissected and fixed at 10% neutral buffered formalin for 24 h. thin sections of tissue around 5 µm, were cut and stained with haematoxylin and eosin. The sections were dehydrated by using increasing concentration of alcohol. They were then treated with diphenyxylene (DPX) and observed under microscope. The observations regarding histological investigation are shown in Fig. 3.

### 2.10 Statistical analysis

The results were expressed as mean SEM from six animals. The results were subjected to statistical analysis by using one way ANOVA followed by Tuckey's test *p* < 0.05 was considered to be statistically significant.
