# Supplementary material for: Data on investigation of hypoglycemic, anti-cholesteremic, in vivo antioxidant and pancreatic beta cell protective effect of Putranjiva roxburghii Wall bark in streptozotocin-induced diabetic rats
Source: Data Brief. 2018 May 2;18:1839–46. doi: 10.1016/j.dib.2018.04.124 (PMC5999010; doi:10.1016/j.dib.2018.04.124)
Supplement: Supplementary file 1 — Supplementary material [file mmc1.pdf]

Data on investigation of hypoglycemic, anticholesteremic, *in vivo* antioxidant and pancreatic beta cell protective effect of *Putranjiva roxburghii* Wall bark in streptozotocin-induced diabetic rats

Kalyani A. Kedar*[1], Sanjay R.Chaudhari[2], Avanapu S. Rao[3]

The Authors declared no conflict of Interest.

1. Corresponding author
   Kedar Kalyani Abhimanyu
   Assistant professor
   Department of Pharmacognosy,
   Progressive Education society's Modern College of Pharmacy,
   Sector -21, Yamunanagar Nigdi, Pune-411044, Maharastra
   Research scholar, Jawaharlal Nehru Technological University (JNTU),
   Hyderabad, Andra Pradesh, India 500072
   +91 9764012744 (m)
   Fax no. 020-27661314.
   E mail id: kk_pharma20@rediffmail .com

2. Dr. Chaudhari Sanjay Ravindra
   Principal & Professor
   Rasiklal M. Dhariwal Institute of Pharmaceutical Education and Research, Pune
   ph:9822299601
   e-mail: dr_srchaudhari@yahoo.com

3. Dr. Rao Avanapu Srinivasa
   Principal & Professor
   Bhaskar Pharmacy College,
   Yeknapally, Moinabad(Mandal)
   R.R(Dt), Hyderabad-500075
   Ph: +91-9704014333(M)
   E-Mail: dravanapu@yahoo.com
