# Supplementary material for: Data on investigation of hypoglycemic, anti-cholesteremic, in vivo antioxidant and pancreatic beta cell protective effect of Putranjiva roxburghii Wall bark in streptozotocin-induced diabetic rats
Source: Data Brief. 2018 May 2;18:1839–46. doi: 10.1016/j.dib.2018.04.124 (PMC5999010; doi:10.1016/j.dib.2018.04.124)
Supplement: Supplementary file 2 — Supplementary material [file mmc2.pdf]

DEPARTMENT OF CHEMISTRY
SAVITIRIBAI PHULE PUNE UNIVERSITY
GCMS REPORT

Sample Information

Analyzed by	: Admin
Analyzed	: 30-04-2015 14:37:19
Sample Type	: Unknown
Level #	: 1
Sample Name	: EA P
Sample ID	: EA P
IS Amount	: [1]=1
Sample Amount	: 1
Dilution Factor	: 1
Vial #	: 1
Injection Volume	: 1.00
Data File	: F:\gcms2015\APRIL\DATA\EA P.qgd
Org Data File	: F:\gcms2015\APRIL\DATA\EA P.qgd
Method File	: F:\gcms2015\APRIL\METHOD\EA P.qgm
Org Method File	: F:\gcms2015\APRIL\METHOD\EA P.qgm
Report File	:
Tuning File	: G:\Toshvin\GCMS IQOQ Data\EI With Column 211014.qgt
Modified by	: Admin
Modified	: 30-04-2015 16:07:02

Chromatogram EA P F:\gcms2015\APRIL\DATA\EA P.qgd

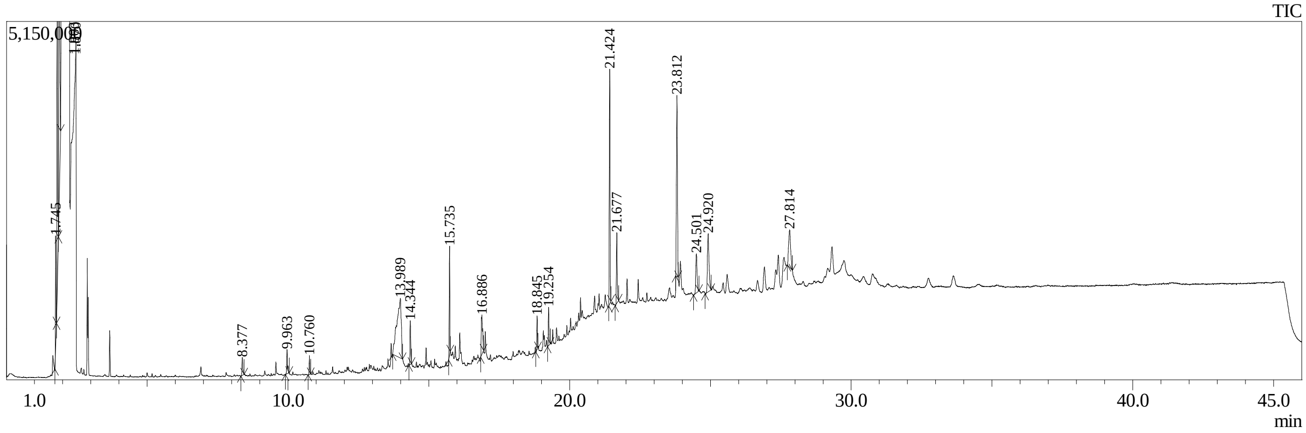

Peak Report TIC

Peak#	R.Time	I.Time	F.Time	Area	Area%	Height	Height%	A/H	Mark	Name
1	1.745	1.725	1.780	2457921	3.84	1672782	6.15	1.47		
2	1.803	1.780	1.845	7681634	12.00	4328487	15.92	1.77		
3	1.870	1.845	1.925	13090020	20.45	6707767	24.67	1.95	V	
4	8.377	8.330	8.445	548554	0.86	258589	0.95	2.12	MI	
5	9.963	9.905	10.040	673382	1.05	349670	1.29	1.93	MI	
6	10.760	10.715	10.810	426627	0.67	272835	1.00	1.56	MI	
7	13.989	13.715	14.070	9161600	14.31	861425	3.17	10.64	MI	
8	14.344	14.290	14.390	1120974	1.75	627707	2.31	1.79	MI	
9	15.735	15.710	15.765	2163029	3.38	1577787	5.80	1.37		
10	16.886	16.845	16.940	1874005	2.93	562673	2.07	3.33	MI	
11	18.845	18.800	18.875	682069	1.07	485467	1.79	1.40	MI	
12	19.254	19.215	19.315	905975	1.42	551296	2.03	1.64	MI	
13	21.424	21.385	21.465	6522289	10.19	3370345	12.40	1.94		
14	21.677	21.620	21.745	2106792	3.29	1015257	3.73	2.08	MI	
15	23.812	23.770	23.860	6789307	10.61	2603621	9.58	2.61		
16	24.501	24.400	24.590	1790970	2.80	561463	2.07	3.19	MI	
17	24.920	24.815	25.030	3347136	5.23	838130	3.08	3.99	MI	
18	27.814	27.730	27.910	2671804	4.17	540416	1.99	4.94	MI	
				64014088	100.00	27185717	100.00			
